# Protection of the Peritoneal Membrane by Peritoneal Dialysis Effluent-Derived Mesenchymal Stromal Cells in a Rat Model of Chronic Peritoneal Dialysis

**DOI:** 10.1155/2019/8793640

**Published:** 2019-09-16

**Authors:** Lan Zhou, Ming Zong, Qiunong Guan, Gerald da Roza, Hao Wang, Hualin Qi, Caigan Du

**Affiliations:** ^1^Department of Urologic Sciences, University of British Columbia, Vancouver, BC, Canada; ^2^Shanghai East Hospital, Tongji University School of Medicine, Shanghai, China; ^3^Division of Nephrology, Department of Medicine, University of British Columbia, Vancouver, BC, Canada; ^4^Department of General Surgery, Tianjin Medical University General Hospital, Tianjin, China; ^5^Tianjin General Surgery Institute, Tianjin, China

## Abstract

Peritoneal dialysis (PD) is a renal replacement option for patients with end-stage renal disease. However, a long-term exposure to hypertonic PD solutions leads to peritoneal membrane (PM) injury, resulting in ultrafiltration (UF) failure. This study was designed to primarily evaluate efficacy of PD effluent-derived mesenchymal stromal cells (pMSCs) in the prevention of PM injury in rats. The pMSCs were isolated from PD effluent. Male Wistar rats received daily intraperitoneal (IP) injection of 10 mL of Dianeal (4.25% dextrose) and were treated with pMSCs (1.2‐1.5 × 10^6^/rat/wk, IP). UF was determined by IP injection of 30 mL of Dianeal (4.25% dextrose) with dwell time of 1.5 h, and PM injury was examined by histology. Apoptosis was quantitated by using flow cytometric analysis, and gene expression by using the PCR array and Western blot. Here, we showed that as compared to naive control, daily IP injection of the Dianeal PD solution for 6 weeks without pMSC treatment significantly reduced UF, which was associated with an increase in both PM thickness and blood vessel, while pMSC treatment prevented the UF loss and reduced PM injury and blood vessels. *In vitro* incubation with pMSC-conditioned medium prevented cell death in cultured human peritoneal mesothelial cells (HPMCs) and downregulated proinflammatory (i.e., CXCL6, NOS2, IL1RN, CCL5, and NR3C1) while upregulated anti-inflammatory (i.e., CCR1, CCR4, IL9, and IL-10) gene expression in activated THP1 cells. In conclusion, pMSCs prevent bioincompatible PD solution-induced PM injury and UF decline, suggesting that infusing back *ex vivo*-expanded pMSCs intraperitoneally may have therapeutic potential for reduction of UF failure in PD patients.

## 1. Introduction

Peritoneal dialysis (PD) is an effective renal replacement therapy for end-stage renal disease, and it has several advantages over hemodialysis (HD) such as cost-effectiveness, better protection of residual renal function and outcomes of transplantation, and lower doses of erythropoietin required [1]. However, ultrafiltration failure (UFF) is a common problem in PD patients, 30-50% of patients having UFF after 6 years of PD [2, 3], and it is one of the main causes for patient dropout from PD therapy [4, 5]. Numerous pathological studies have demonstrated a close association of UFF with peritoneal membrane (PM) injury or structural alterations (e.g., persistent injury/inflammation, fibrosis, and neoangiogenesis) [6, 7]; therefore, successful protection of the PM structure or reduction of PM damage may result in prolongation of PD therapy.

Mesenchymal stromal cells (MSCs) are multipotent fibroblast-like adult cells that were firstly isolated from the bone marrow (BM) by Friedenstein and his colleagues in 1974 [8] and have recognized capacities for both self-renewal and multilineage differentiation potential [9, 10]. Due to the multipotency and paracrine effects as well as anti-inflammation or immunomodulation of these cells, MSCs have been widely considered an ideal candidate for the development of a novel cell therapy for both tissue repair [11] and immunosuppression or anti-inflammation [12, 13]. Interestingly, several experimental studies have showed the efficacy of MSCs, either from rats or from human umbilical Wharton's jelly, in the reduction of tissue injury (submesothelial thickness), inflammation, angiogenesis, and fibrosis of PM, resulting in the prevention of UFF in rat models of PD [14]. Very recently, a nonrandomized, placebo-free, phase I clinical trial shows that infusion of autologous adipose tissue-derived MSCs (AD-MSCs) in PD patients with expected peritoneal fibrosis significantly decreases the rate of solute transport (creatinine) across PM with no serious adverse events and no catheter-related complications [15]. We have for the first time isolated MSCs from the otherwise discarded PD effluents from PD patients [16]. This type of MSCs expresses some unique cell surface markers, such as negative markers in the expression of CD105, Strol-1, and SSE-4 and positive markers in CD200 [16, 17], indicating the difference from those common MSCs (i.e., MSCs from BM, umbilical cords, or adipocytes) [10, 18, 19]. To evaluate the possibility of using these PD effluent-derived MSCs (pMSCs) to control PM inflammation and repair PM injury in PD patients—autotransplantation of their own pMSCs, we tested the therapeutic potential of pMSCs for protection of PM from functional and structural derangements induced by chronic PD fluid exposure in rats in this study.

## 2. Materials and Methods

### 2.1. Animals and Cells

Male Wistar albino rats (12-14 weeks old, weighing 340–350 g) were purchased from Charles River Laboratories International, Inc. (Wilmington, MA, USA) and were used for the experiments in this study under the animal use protocol approved by the Animal Experiments Ethical Committee of the University of British Columbia following the guideline of the Canadian Council on Animal Care.

Human peritoneal mesothelial cells (HPMCs) were isolated from PD effluents and were grown in K1 complete culture medium (K1 medium) as described previously [20]. THP1 cells (human monocytes) were purchased from Cedarlane Co. (ATCC TIB-202, Burlington, ON, Canada) and were grown in RPMI-1640 medium containing 10% of fetal bovine serum (FBS). Both types of cells were expanded and used for the experiments in a 5% CO_2_ humidified incubator at 37°C.

### 2.2. Preparation of pMSCs for *In Vivo* Treatments and pMSC-Conditioned Medium for *In Vitro* Tests

pMSCs were isolated from PD effluents donated by anonymized patients who were on PD therapy with either Dianeal or Physioneal PD solution within 4 weeks as described previously [16]. This procedure was approved by the Clinical Research Ethics Board at the University of British Columbia (Vancouver, BC) in accordance with the Canadian Tri-Council Policy Statement: ethical conduct for research involving humans (protocol number H15-02466).

pMSCs were expanded in a human protein culture medium (xeno-free medium) in plastic culture dishes as described previously [17]. After four passages, the pure pMSCs were frozen with 10% dimethylsulfoxide (DMSO) in liquid nitrogen. To prepare a large quantity of pMSCs for *in vivo* treatments, frozen vials of cells (approximately 10^6^ cells/vial) were rapidly thawed in a 37°C water bath for 1 min, followed by washing once with the culture medium. The washed cells were grown in the xeno-free medium in the plastic petri dishes in a 5% CO_2_ humidified incubator at 37°C. Then, the medium was changed every 3 days until the cell culture reached 70–80% confluence. Cells were detached by trypsinization and were washed twice with phosphate-buffered saline (PBS). Finally, a dose of 1.2‐1.5 × 10^6^ pMSCs in 1 mL of PBS was prepared and was administered by intraperitoneal (IP) injection to each rat immediately.

For preparation of pMSC-conditioned medium (pMSC-CM), the frozen cells after thawing were grown to be confluent in cultures as described above, followed by incubation with xeno-free medium (approximately 5 mL per 10^6^ cells) in the plastic dishes. After 24 h of incubation, the medium as pMSC-CM was harvested, and its cell debris was removed by centrifugation at 12,000 × g for 10 min at 4°C prior to experimental use.

### 2.3. A Rat Model of Chronic PD and Experimental Groups

Wistar rats received daily 10 mL IP injection of a conventional glucose-based PD solution (Dianeal 4.25% dextrose, 484 mOsmol/L, pH 5.2) (Baxter Healthcare, IL, USA) for a period of 6 weeks, by which PM injury was induced similar to the situation in PD patients. The injection was made using a 22-gauge needle through the middle abdomen as described previously [21, 22].

Rats were randomly divided into three groups: (1) control group—rats (*n* = 3) did not receive any solution injection during the study; (2) vehicle group—rats (*n* = 9) received daily IP injection of the PD solution and were treated with PBS vehicle (IP, 1 mL/rat/wk, starting at day 1); and (3) pMSC group—rats (*n* = 9) received the daily IP injection of the PD solution and were treated with pMSCs (IP, 1.2‐1.5 × 10^6^ cells/rat/wk, starting at day 1). The IP injection of the PD solution was performed in the early morning (9 am–10 am), and the treatment with pMSCs or vehicle in the late afternoon (5 pm–6 pm).

### 2.4. Measurement of Ultrafiltration

Ultrafiltration (UF) or peritoneal fluid transport was determined as a primary outcome of pMSC-based therapy in this study, and it was one of the two common measures of the peritoneal permeability function or UFF in patients [3, 23]. In brief, 30 mL of Dianeal (4.25% dextrose) was slowly injected into the peritoneal cavity of each rat using a 22-gauge needle. After 90 min of dwell time, the dialysate in the peritoneal cavity was collected using a syringe as much as possible, and the recovered volume (mL) represented UF.

### 2.5. Histological Assessment

After dialysate collection and animal euthanasia, two pieces of the anterior parietal peritoneum were harvested from the side opposite to the injection sites, followed by formalin (10%) fixation and paraffin embedding. Tissue sections (4 *μ*m) were stained with hematoxylin and eosin (H&E). All of the tissue sections were scanned using a Leica SCN400 slide scanner (Leica Microsystems Inc., Concord, ON, Canada), and the images were examined by using the Digital Image Hub—A Slidepath Software Solution (Leica Microsystems Inc.) in a blinded fashion. The submesothelial thickness (from the inner surface of the muscle to the mesothelium) and the presence of the blood vessels including capillaries (neoangiogenesis) within the submesothelial layer were determined as previously described [21].

### 2.6. Quantitation of Cell Apoptosis

The cell apoptosis was quantitatively determined by using a fluorescent-activated cell sorting (FACS) technique with double staining of Annexin V conjugated with phycoerythrin (Annexin-V-PE) and 7-amino-actinomycin D (7-AAD) as described in our previous study [24].

### 2.7. Measurement of Cytoprotection of pMSC-CM *In Vitro*

First, the cytoprotective activity of pMSC-CM was measured against oxidative stress- (H_2_O_2_) induced cell death. HPMCs (2 × 10^5^ cells/well) were seeded in K1 medium in 24-well plates overnight, followed by four different treatments: (1) 1 mL/well of xeno-free medium only (medium group), (2) 1 mL/well of xeno-free medium containing 0.1 *μ*m of H_2_O_2_ (H_2_O_2_ group), (3) 1 mL/well of xeno-free medium containing 0.1 *μ*m of H_2_O_2_ and 25% of pMSC-CM (*v*/*v*) (H_2_O_2_+25% group), and (4) 1 mL/well of xeno-free medium containing 0.1 *μ*m of H_2_O_2_ and 50% of pMSC-CM (*v*/*v*) (H_2_O_2_+50% group). The apoptosis or viability was counted by FACS analysis after 24 h of incubation in a 5% CO_2_ incubator at 37°C.

Second, the cytoprotective activity of pMSC-CM was determined in the environment of direct exposure to a hypertonic PD solution (PD solution-induced cell death). The monolayer of HPMCs after overnight incubation with K1 medium was treated with xeno-free medium only (medium group) or with a peritoneal dialysis solution (Dianeal 4.25% dextrose, denoted as PDS) for 25 min. Subsequently, these PDS-treated cells were incubated with the xeno-free medium (PDS group) or with the xeno-free medium containing 50% (*v*/*v*) pMSC-CM (PDS+50% group) in a 5% CO_2_ incubator at 37°C. After 24 h or 48 h of treatment, the apoptosis was determined by FACS analysis.

### 2.8. Inactivation of Monocytes/Macrophages by pMSC-CM *In Vitro*

THP1 monocytes (approximately 10^5^ cells/mL) were activated or induced to macrophage differentiation by initial stimulation with 100 ng/mL of phorbol 12-myristate 13-acetate (PMA) for 24 h, followed by additional stimulation with 2 ng/mL of lipopolysaccharides (LPS) for 24 h in a CO_2_ incubator at 37°C. The activated THP1 cells were then treated with xeno-free medium (PMA/LPS group) or the xeno-free medium containing 50% (*v*/*v*) of pMSC-CM (PMA/LPS+50%) for 24 h. The unstimulated THP1 cells after 24 h incubation with the xeno-free medium were used as a baseline control (control group).

### 2.9. RNA Extraction

After 24 h of treatment with pMSC-CM or medium control, total RNA was extracted from THP1 cells by using a mirVana™ isolation kit (Ambion, Austin, TX, USA). Only the RNA samples with RNA integrity number (RIN) ≥ 8 were used for PCR array analysis as described below.

### 2.10. PCR Array Analysis

The expression of a panel of monocyte-/macrophage-expressing genes in THP1 cells was examined by using a PCR array following the manufacturer's instruction (SABiosciences—QIAGEN Inc., Valencia, CA, USA), and each group represented three separate samples. In brief, 1 *μ*g of high-quality total RNA from each sample was reverse transcribed to cDNA by using the RT^2^ First Strand Synthesis Kit (QIAGEN). The expression of selected genes was then amplified by real-time PCR using RT^2^ Profile PCR arrays with PCR amplification conditions (10 min at 95°C, followed by 45 cycles of 15 s at 95°C and 60 s at 60°C) according to the manufacturer's instruction (QIAGEN Inc.). The fold change of each target transcript and statistical comparison between groups were determined by using the manufacturer's online web analysis tools (http://www.SABiosciences.com/pcrarraydataanalysis.php).

### 2.11. Nitric Oxide (NO) Measurement

After 24 h of treatment with pMSC-CM or medium control, the levels of nitrite (a product of NO by oxidation) in the supernatant of THP1 cell cultures were measured using the Griess method. In brief, 50 *μ*L of culture supernatant was first incubated with 50 *μ*L of 1% sulfanilamide in 5% phosphoric acid (96-well plates in triplicate) for 10 min, followed by the addition of 50 *μ*L/well of 0.1% naphthylethyline diamine dihydrochloride. The color development was quantitatively measured at 550 nm, and the level of NO/nitrite in each sample was calculated using a standard curve with known sodium nitrite concentrations.

### 2.12. Western Blot

After 24 h of treatment with pMSC-CM or medium control, total cellular protein from THP1 cells was harvested and the protein levels of nitric oxide synthase 2 (NOS2) were determined by Western blot as described previously [25]. Briefly, protein samples (approximately 100 *μ*g protein/sample) were fractionated by 7% SDS-polyacrylamide gel electrophoresis (SDS-PAGE) and then were transferred onto a nitrocellulose membrane. NOS2 protein bands were specifically detected by a primary rabbit polyclonal anti-NOS2 antibody (N-20) (Santa Cruz Biotech, Santa Cruz, CA, USA) and secondary goat anti-rabbit IgG antibody (Vector Lab., Burlingame, CA, USA). Blots were reprobed using anti-glyceraldehyde 3-phosphate dehydrogenase (GAPDH) (Epitope Biotech Inc., Vancouver, BC) for confirmation of loaded protein in each sample. The expression levels of NOS2 proteins were measured using densitometry and were presented as a ratio unit (RU) of the target protein to GAPDH on the same blots.

### 2.13. Statistical Analysis

Statistical analysis was performed by using GraphPad Prism software (GraphPad Software, Inc., La Jolla, CA, USA). Data were collected from each individual experiment or each rat and were compared between groups by using analysis of variance (ANOVA) or *t*-tests (two-tailed distribution) as appropriate. A *P* value of ≤0.05 was considered significant.

## 3. Results

### 3.1. Isolation and Culture of pMSCs from PD Effluents

**Figure 1:**
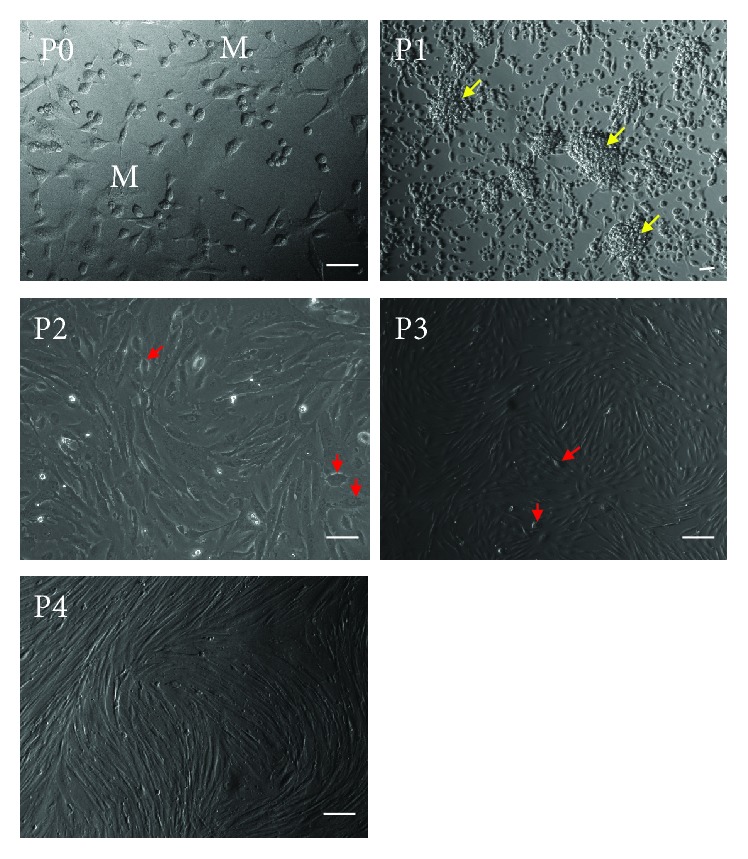
Isolation and culture of pMSCs. Peritoneal cells from PD effluents were grown in the xeno-free medium in plastic petri dishes, followed by medium change once every 2-3 days. Data represented a typical microscopic image of different stages (passages) of cell cultures. P0: seeding peritoneal cells after removal of nonadherent cells by changing the culture medium at the beginning of pMSC culture. Red “M”: macrophages. P1: cells on passage 1 of culture. Yellow arrows: MSC colonies. P2 or P3: fibroblast-like cells on passage 2 or 3 of culture. Red arrows: “cobblestone”-like mesothelial cells. P4: a homogenous monolayer of spindle-shaped pMSCs in culture. Scale bar: 100 *μ*m.

After pelleting from PD effluents by centrifugation, cells were resuspended in and grown with the xeno-free medium in a plastic culture dish. The culture medium was changed once every 2-3 days afterward. As shown in Figure 1, there were many macrophages in the beginning of cell cultures (P0, seeding cells) and pMSCs were not distinguishable from peritoneal mesothelial cells in the morphology. At the end of the first passage of cell culture (P1), pMSCs formed distinct MSC colonies. These colonies were expanded and formed a monolayer of adherent fibroblast-like cells on passages 2 to 3 (P2 and P3) of cell cultures, while some cobblestone-like mesothelial cells were sill noticeable under a microscope. On passage 4 (P4), the adherent fibroblast-like cells formed a homogenous monolayer of spindle-shaped pMSCs in the culture, indicated by the unique characteristics of MSCs.

### 3.2. Treatment with pMSCs Prevents the Loss of Peritoneal UF

**Figure 2:**
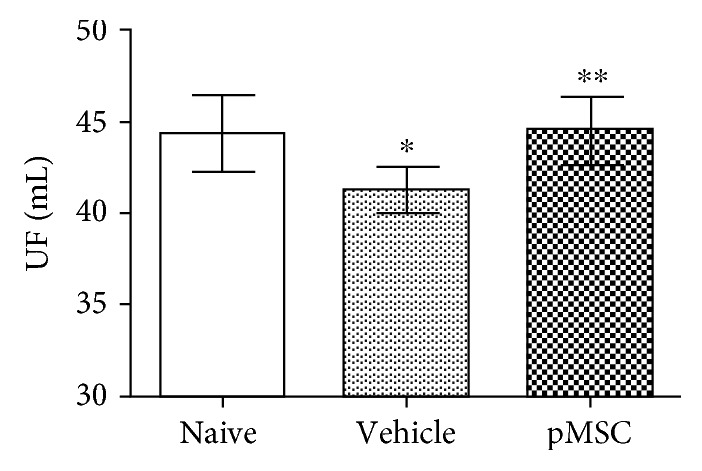
Treatment with pMSCs prevents loss of peritoneal ultrafiltration (UF). The peritoneum of Wistar rats was daily exposed to Dianeal (4.25% dextrose) and was treated with either vehicle PBS or pMSCs. The UF as a parameter of peritoneal function was measured at the end of 6 weeks of treatment by using Dianeal (4.25% dextrose) with dwell time of 90 min. Data were presented as mean ± standard deviation (SD) of each group (naïve: *n* = 3, vehicle: *n* = 9, and pMSCs: *n* = 9) and were statistically analyzed using the two-tailed *t*-test. ^∗^*P* = 0.0091 (vehicle vs. naïve), ^∗∗^*P* = 0.0005 (pMSCs vs. vehicle), and *P* = 0.8971 (naïve vs. pMSCs).

UFF develops over time on PD, and it becomes the main reason for the abandonment of this therapy [2, 3]. In rats, daily intraperitoneal exposure to a hypertonic PD solution (Dianeal 4.25%) for 6 weeks significantly reduced UF from 44.33 ± 2.08 mL in the naïve control group to 41.25 ± 1.22 mL in the vehicle group (*P* = 0.0091) (Figure 2). Treatment with pMSCs then significantly prevented the loss of the UF, indicated by the fact that UF (44.5 ± 1.88 mL) is higher in the pMSC-treated group than in the vehicle group (pMSC-treated vs. vehicle, *P* = 0.0005), and there was not significant difference in UF between the control and the pMSC-treated group (*P* = 0.8971) (Figure 2).

### 3.3. Treatment with pMSCs Reduces PM Injury

**Figure 3:**
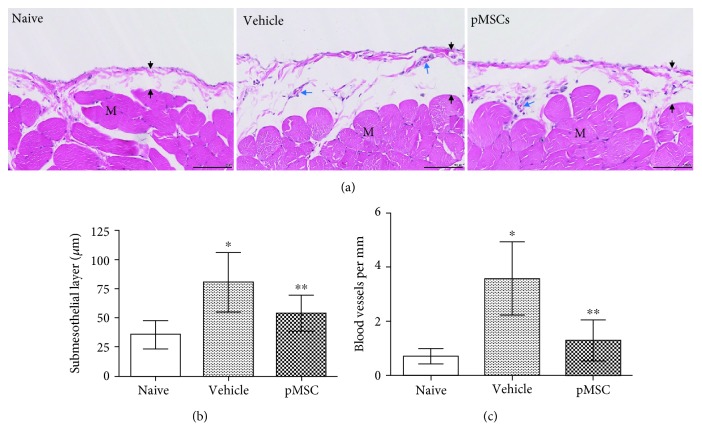
Treatment with pMSCs prevents an increase in submesothelial layer thickening and neoangiogenesis of the peritoneal membrane (PM). At the end of 6 weeks of treatment, peritoneal tissue sections (2 sections/rat) were taken from rats (naïve: *n* = 3, vehicle: *n* = 9, pMSCs: *n* = 9) after UF measurement. (a) A typical microscopic image of H&E-stained peritoneal tissue sections in each group, showing the thickness of the submesothelial layer of the PM indicated by the distance between black two arrows. Nuclear dark blue stain: cellular infiltrates; blue arrows: blood vessels; black thin bar: 100 *μ*m. M: muscle. (b) The thickness of the submesothelial layer in each tissue section was measured using the Digital Image Hub software. Data were presented as mean ± SD of each group and were statistically analyzed using the two-tailed *t*-test. ^∗^*P* = 0.0164 (vehicle vs. naïve), ^∗∗^*P* = 0.0168 (pMSCs vs. vehicle), and *P* = 0.0958 (naïve vs. pMSCs). (c) The density of blood vessels (neoangiogenesis) in the PM was counted as the number of the blood vessels and capillaries per millimeter. Data were presented as mean ± SD of each group and were statistically analyzed using the two-tailed *t*-test. ^∗^*P* = 0.0053 (vehicle vs. naïve), ^∗∗^*P* = 0.0004 (pMSCs vs. vehicle), and *P* = 0.2374 (naïve vs. pMSCs).

A further histological analysis revealed a close association of the reduction of UF with the severity of PM structural alterations—increased thickness and angiogenesis of the submesothelial layer among these groups (Figure 3). As compared with control rats, the submesothelial layer of rats receiving IP injection of the PD solution became thicker, indicated by 80.4 ± 25.24 *μ*m in the vehicle group compared to 35.85 ± 12.0 *μ*m in the control group (*P* = 0.0164) (Figure 3(b)). The PD solution-induced PM thickness was effectively prevented by pMSC treatment, indicated by the thinner submesothelial layer of pMSC-treated rats (54.06 ± 15.47 *μ*m) than that of untreated rats in the vehicle group (*P* = 0.0168) and by no significant difference from that of the control rats (*P* = 0.0958) (Figure 3(b)). Similar alteration of angiogenesis—blood vessel numbers—within the submesothelial layer of rats among these groups was observed. There were more blood vessels in the vehicle group (3.589 ± 1.355) than those in the control group (0.705 ± 0.285, *P* = 0.0053) or in the pMSC-treated group (1.283 ± 0.758, *P* = 0.0004), suggesting that pMSC treatment effectively prevented PD solution-induced angiogenesis within the PM.

### 3.4. pMSC-CM Prevents Cell Death of Cultured HPMCs

**Figure 4:**
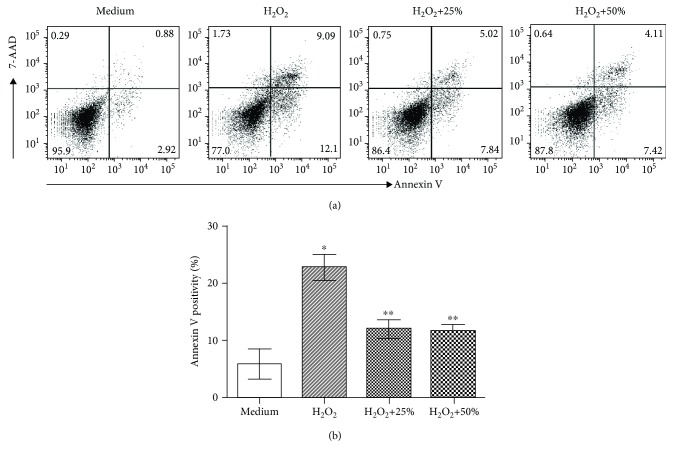
Incubation with pMSC-conditioned medium (pMSC-CM) inhibits H_2_O_2_-induced apoptosis in cultured human peritoneal mesothelial cells (HPMCs). HPMCs (2 × 10^5^ cells/well) were grown in 24-well plates overnight, followed by treatment with 0.1 *μ*M H_2_O_2_ in the absence or presence of 25% or 50% (*v*/*v*) of pMSC-CM for 24 h. Cell apoptosis was determined by flow cytometric analysis with Annexin-V-PE and 7-AAD staining. (a) A typical dot plot showing the percentage of Annexin-V-PE- and/or 7-AAD-positive staining of cell populations in each group. (b) Apoptosis was measured by the sum of Annexin V-stained cell populations (Annexin-V-PE positive and Annexin-V-PE and 7-AAD double positive in the right lower and upper quadrants). Data are presented as mean ± SD of seven determinants and were statistically analyzed by using the two-tailed *t*-test. ^∗^*P* < 0.0001 (H_2_O_2_ vs. medium) and ^∗∗^*P* < 0.0001 (pMSCs+25% or 50% vs. H_2_O_2_).

To verify the direct cytoprotection of pMSCs against PM injury, the effect of pMSC-CM on cell death of cultured HPMCs was examined. As shown in Figure 4, the addition of 0.1 *μ*M H_2_O_2_ for 24 h significantly induced cell apoptosis (Annexin V positivity) in HPMCs from 5.82 ± 2.59% in untreated controls to 22.71 ± 2.23% in H_2_O_2_-treated cultures (*P* < 0.0001). The H_2_O_2_-induced cell death was significantly reduced to 11.98 ± 1.65% in the H_2_O_2_-treated cultures in the presence of 25% (*v*/*v*) of pMSC-CM or to 11.67 ± 1.07% in the presence of 50% (*v*/*v*) of pMSC-CM (*P* < 0.0001, H_2_O_2_ vs. H_2_O_2_+25% or H_2_O_2_+50%).

**Figure 5:**
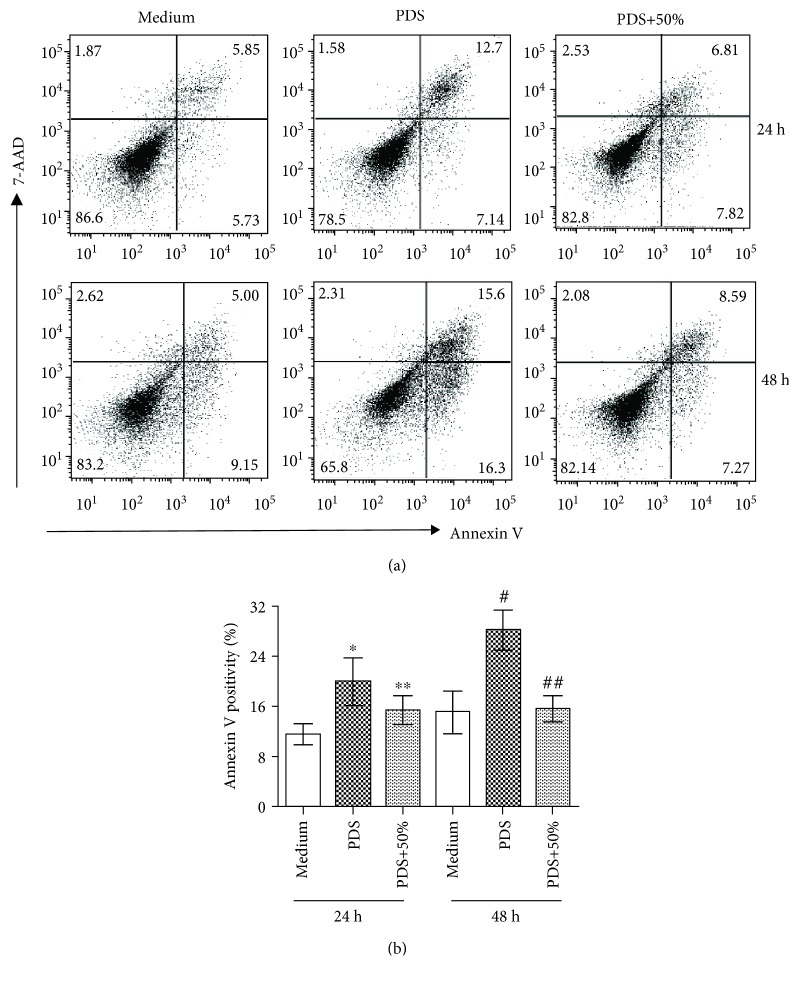
Incubation with pMSC-CM inhibits hypertonic peritoneal dialysis solution- (PDS-) induced apoptosis in cultured HPMCs. HPMCs (2 × 10^5^ cells/well) were grown in 24-well plates overnight and were briefly exposed to Dianeal (4.25% dextrose), followed by being cultured in the xeno-free medium in the absence or presence of 50% (*v*/*v*) of pMSC-CM for 24 h or 48 h. Cell apoptosis was determined by flow cytometric analysis with Annexin-V-PE and 7-AAD staining. (a) A typical dot plot showing the percentage of Annexin-V-PE- and/or 7-AAD-positive staining of cell populations in each group. (b) The sum of Annexin V-stained cell populations as total apoptosis in each group. Data are presented as mean ± SD of each group (medium: *n* = 5, PDS: *n* = 3, and PDS+50%: *n* = 3) and were statistically analyzed by using the two-tailed *t*-test. ^∗^*P* = 0.0021 (PDS vs. medium), ^∗∗^*P* = 0.0740 (PDS+50% vs. PDS), ^#^*P* = 0.0008 (PDS vs. medium), and ^##^*P* = 0.0022 (PDS+50% vs. PDS).

The antiapoptotic activity of pMSC-CM was further evaluated in cultured HPMCs after a brief exposure to a hypertonic PD solution. HPMC monolayers were treated with a Dianeal solution (4.25% glucose, pH 5.2) for 25 min, followed by reculturing the cells in the normal culture medium. As shown in Figure 5, a brief exposure to the PD solution induced a significant amount of cell apoptosis, 19.94 ± 3.78% from 11.52 ± 1.85% (untreated control) after 24 h or 28.19 ± 3.17% from 15.11 ± 3.39% (untreated control) after 48 h. Again, the addition of 50% of pMSC-CM significantly reduced the cell apoptosis to 15.38 ± 2.28% (*P* = 0.0740, PDS vs. PDS+50%, after 24 h) and 15.8 ± 2.03% (*P* = 0.0022, PDS vs. PDS+50%, after 48 h).

### 3.5. pMSC-CM Inhibits Inflammatory Responses of Activated Macrophages

To investigate the anti-inflammatory activities of pMSCs, the effect of pMSC-CM on the activation of macrophages was examined by PCR array analysis of the expression of a panel of 77 inflammation-related gene transcripts (Suppl. [Supplementary-material supplementary-material-1]). Stimulation of THP1 cells with PMA/LPS upregulated the expression (>1.5-fold as compared to control) of total 28 gene transcripts, including CXCL8 (IL8), IL1B, CXCL6, IL1RN, NOS2, RIPK2, BCL6, and ITGB2 with a *P* value of <0.05, and of CCL5 and TNF in a trend toward significance (Table 1). The addition of 50% pMSC-CM to PMA/LPS-stimulated cells suppressed the expression of all of these 28 gene transcripts (from -1.03- to -10.08-fold) (Suppl. [Supplementary-material supplementary-material-1]). The downregulation of CXCL6, NOS2, IL1RN, CCL5, and NR3C1 transcripts was statistically significant (Table 1), and that of IL8, RIPK2, BCL6, ITGB2, and MYD88 transcripts less significant (Table 1). Interestingly, pMSC-CM also significantly stimulated the expression (>1.5-fold as compared to PMA/LPS) of a group of anti-inflammatory gene transcripts, such as CCR1, CCR4, IL9, and IL-10, in activated macrophages with a *P* value of <0.05 (Table 1).

**Figure 6:**
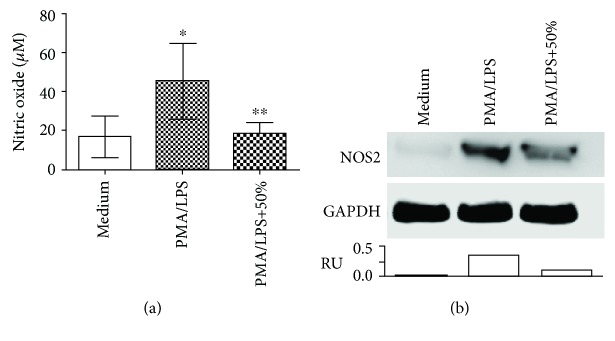
Incubation with pMSC-CM inhibits the expression of NOS2 in activated THP1 cells. PMA/LPS-activated THP1 cells were incubated in the absence or presence of 50% pMSC-CM (*v*/*v*) for 24 h, while unactivated cells were used as the medium control. (a) NO production in THP1 cell cultures in each group. Data were presented as mean ± SD of four determinants and were statistically analyzed using the two-tailed *t*-test. ^∗^*P* = 0.0410 (PMA/LPS vs. medium), ^∗∗^*P* = 0.0367 (PMA/LPS+50% vs. PMA/LPS), and *P* = 0.8203 (PMA/LPS+50% vs. medium). (b) Western blot analysis of cellular NOS2 protein expression in each group. Data represented a typical Western blot of NOS2 protein of three separate examinations. RU: ratio unit of NOS2 to GAPDH.

To confirm the downregulation of NOS2 mRNA in activated THP1 cells shown by PCR array analysis, the nitric oxide (NO) produced by NOS2 and its protein levels were examined in PMA/LPS-activated THP1 cells in the absence or presence of 50% of pMSC-CM. As expected, PMA/LPS significantly stimulated NO production from 16.74 ± 10.7 *μ*M (medium only) to 45.4 ± 19.34 *μ*M (*P* = 0.0410), which was reduced to 18.21 ± 6.24 *μ*M in the presence of 50% pMSC-CM (*P* = 0.0367, PMA/LPS vs. PMA/LPS+50%) (Figure 6(a)). In parallel, the expression of NOS2 protein was upregulated by PMA/LPS stimulation, and its upregulation was suppressed by the pMSC-CM in the Western blot assay (Figure 6(b)). These data confirmed the changes of NOS2 transcript from the PCR array analysis.

## 4. Discussion

The therapeutic potential of MSCs and their products (e.g., extracellular vesicles or secretome) has been actively evaluated for tissue damage repair, immunomodulation, and anti-inflammation in many different pathologies for more than a decade [26, 27], including the peritoneal inflammation and fibrosis [14]. However, the clinical use of these MSCs is largely limited by their source, ethical issue, and safety concern from patients. We for the first time identified MSCs in otherwise “discarded” PD fluids—pMSCs [16], and thought that it may be possible to use patients' own pMSCs to control their PM inflammation or even repair their PM injury. Unlike BM-MSCs or AD-MSCs, these pMSCs express CD200 but not CD105 [17]. In the present study, the biological functions of pMSCs were investigated for the first time. IP injection of pMSCs significantly protected PM from a Dianeal PD solution- (4.25% glucose) induced morphological alteration, neoangiogenesis, and functional loss (fluid removal) in a rat model of chronic PD. Also, like other sources of MSCs, pMSCs secreted bioactive factors in cultures that could protect peritoneal mesothelial cells from apoptosis and inactivate the inflammatory response of activated macrophages, including downregulation of NOS2 expression.

The UF of PD provides both solute clearance and fluid removal for PD patients, and UFF in either or both of these parameters is one of the main causes of PD technique failure, resulting in transferring to HD [4, 28]. As of today, the mechanisms underlying UFF are not completely understood, but the pathological changes of peritoneal biopsies from PD patients indicate that UFF is associated with the PM alterations such as an increase in macrophage infiltration [29], thickness or fibrosis of the submesothelial layer [30–32], and neoangiogenesis [29, 32, 33]. Similarly in rats, a long term of peritoneal exposure to a hypertonic PD solution leads to mesothelial cell damage, fibrosis or increased thickness of PM, and neoangiogenesis, resulting in an increase in lymph flow or loss of fluid removal [34–36]. Therefore, any therapy that targets these alterations of the PM will prolong the UF in PD patients.

Recently, there has been an increasing interest in using MSCs to protect the PM of PD patients from injury during PD. IP injection of rat BM-MSCs or AD-MSCs significantly reduces PD solution-induced submesothelial thickness and cellular infiltration of the PM [37, 38] as well as chlorhexidine gluconate-induced peritoneal fibrosis in rats [39, 40]. Similar to the results from this study, treatment with human umbilical cord-derived MSCs prevents methylglyoxal/PD solution-induced peritoneal alterations including peritoneal thickening, fibrosis, and neoangiogenesis, which is associated with the protection of UF (both fluid removal and glucose and creatinine clearance) [41]. Taken together, these findings may provide preclinical evidence for a novel approach by using MSCs in the treatment of PM alterations in PD patients.

There are five different pathways mediating MSC functions: (1) differentiation into cell types in target tissues; (2) fusion with dying cells for tissue rescue or repair; (3) paracrine secretion of soluble factors, such as stromal-derived factor 1 (SDF-1), hepatocyte growth factor (HGF), TNF-*α*-stimulated gene protein 6 (TSG-6), and prostaglandin E2 (PGE2), to promote tissue rescue or repair; (4) transfer of organelles (e.g., mitochondria); or (5) bioactive substances from MSCs to target cells via tunneling nanotubes or extracellular vehicles (e.g., exosomes) [42]. However, whether all of these pathways or just some of them are required for the MSC functions in the reduction or repair of the peritoneum injury remains to be further investigated. IP injected MSCs can be localized in the mesothelium line [37], but only until day 3 [39], and by then, these MSCs are colocalized but not merged with injured mesothelial cells [38]. In the present study, we did not specifically track the migration and/or possible differentiation of pMSCs after IP injection in rats. However, at the experimental endpoint of this study (at day 7 after the last injection), we did not find any of injected human cells in the PM sections by immunohistochemical staining of human nuclear antigen (data not shown), suggesting that it was possible that these human cells were eliminated by the rat immune responses against xenoantigens on pMSCs. Taken together, all these studies may suggest that the beneficial effects of MSC-based therapy on peritoneal injury are most likely associated with their paracrine secretion or direct transfer of cytoprotective substances including extracellular particles during the temporary retention in the peritoneal cavity after IP injection.

Indeed, the culture supernatant or the secretome from pMSCs (pMSC-CM) protects peritoneal mesothelial cells from oxidative stress (H_2_O_2_) or hypertonic PD solution-induced cell death (Figures 4 and 5). Other studies in literature have demonstrated that MSC-CM at least in part by secretion of HGF inhibits TGF-*β*1-induced epithelial-mesenchymal transition of HPMCs [39] and/or stimulates mesothelial cell proliferation [38]. It has also been known that the numbers of infiltrating macrophages in the parietal peritoneum biopsies from the predialysis stage are specifically associated with higher PD technique failure and mortality in PD patients [29, 43], indicating a key role of macrophages in the initiation of peritoneal inflammation in PD patients. There are two pathways of MSCs specifically for inactivation of macrophages. One is the secretion of PGE2 and others (e.g., tumor growth factor-*β*3, thrombospondin 1, and IL-6) that drives resident macrophages with an M1 proinflammatory phenotype toward an M2 anti-inflammatory phenotype for inflammation suppression or tissue repair [44–46]. The other is the production of TSG-6 that binds to CD44 on resident macrophages, resulting in the disruption of TLR2/NF-*κ*B signaling and the decrease in the secretion of proinflammatory mediators [44]. The culture supernatant from pMSCs inactivates macrophages by downregulation of many inflammatory gene expression markers including NOS2, an M1 phenotype marker (Table 1, Figure 6), suggesting that pMSCs may also secret these soluble factors, which however remains to be further investigated—the composition of the secretome from pMSCs. It has been considered that the secretome is a primary means by which MSCs conduct many of their therapeutic effects [47].

The limitation of this study was that it was largely experimental study-related. First, the pathogenesis of PM injury in rats may not be the same as in PD patients due to the differences of genome, anatomy of the peritoneum, physiology (e.g., uremia in PD patients), and the period of exposure to the PD solutions between experimental rats and PD patients. Second, the possible stimulation of xenoimmunity by repeated IP injection of human pMSCs in rats may negatively affect their therapeutic actions in vivo. Third, human pMSCs may exhibit different biological functions in different hosts—rats versus humans, particularly in the situation of using pMSCs as cell autotransplantation. All these suggest that the efficacy of pMSCs in the protection of PM needs further evaluation in different PD models, such as in uremic rats. In addition, the molecular mechanisms underlying the cytoprotection and immunomodulation of pMSCs remain to be further investigated, for example, which secreted molecules from pMSCs protect mesothelial cells from apoptosis and which inactivate macrophages or switch the macrophage phenotype from M1 (an inflammatory mediator) to M2 (a tissue healing mediator).

## 5. Conclusion

PM injury or structural alterations including persistent inflammation, fibrosis, or neoangiogenesis and subsequently functional loss (i.e., UFF) of PM develop in PD patients over time on PD therapy [6, 7]. Unfortunately, clinically available treatment for UFF is limited at present. MSCs are promising therapeutic cells for tissue injury repair and inflammation control [11–13]. The present study demonstrates that IP transplantation of pMSCs that can be isolated from otherwise “discarded” PD fluid ameliorates PD fluid-induced peritoneal damage in rats, suggesting that pMSCs are a potential candidate for MSC autotransplantation (just infusing back the patient's own MSCs) to treat PM injury in PD patients. Further studies are warranted to define their mechanisms (e.g., secretome composition) underlying the cytoprotection of the mesothelium and the suppression of inflammatory responses, particularly macrophage activation in the PM.

## Figures and Tables

**Table 1:** Inactivation of inflammatory responses of macrophages by pMSC-CM.

Affected gene transcripts	Fold changes by PMA/LPS (vs. control)	*P* value (PMA/LPS vs. control, *n* = 3)	Fold changes by pMSC-CM (vs. PMA/LPS)	*P* value (PMA/LPS+50% vs. PMA/LPS, *n* = 3)
CXCL8	535.95	0.009573	-9.14	0.083365
IL1B	500.8	0.048456	-6.25	0.123176
CXCL6	242.74	0.001276	-10.08	0.032372
CXCL3	190.02	0.038563	-2.32	0.133338
IL1RN	177.15	0.004863	-4.02	0.037209
NOS2	11.17	0.007915	-9.35	0.008181
RIPK2	6.02	0.013444	-5.35	0.058044
BCL6	5.27	0.029664	-2.39	0.085688
CCL5	3.8	0.065136	-8.77	0.017685
ITGB2	2.97	0.036245	-2.54	0.056093
NR3C1	2.45	0.234157	-7.81	0.043945
MYD88	2.82	0.092337	-4.90	0.058324
TNF	1.98	0.052401	-1.46	0.140847
CCR1	-1.27	0.428263	1.74	0.026517
CCR4	1.18	0.48383	1.92	0.01117
CXCL9	-1.67	0.138019	1.97	0.000092
CXCR1	-1.11	0.662785	1.60	0.035095
IL10	-1.38	0.309707	1.93	0.015066
IL23A	1.12	0.851924	1.60	0.016877
IL6R	-1.36	0.179679	1.65	0.004205
IL9	-1.25	0.522482	1.93	0.024161
KNG1	-1.25	0.320858	1.80	0.01354
SELE	-1.2	0.477778	1.86	0.005707
TLR3	-1.26	0.363668	2.17	0.008944
TLR5	-1.38	0.298214	1.86	0.036134

The regulatory effects (both upregulation and downregulation) of pMSC-CM on the expression of inflammation-related gene transcripts in PMA/LPS-activated THP1 cells were examined by using the PCR array. The fold change of each transcript level in the experimental group as compared to the control group (PMA/LPS vs. control or PMA/LPS+50% pMSC-CM vs. PMA/LPS) was presented as mean of three separate RNA samples (*n* = 3). A negative number represented downregulation.

## Data Availability

The data used to support the findings of this study are available from the corresponding author upon request.

## Supplementary Materials

Supplementary Materials Supplementary Table 1: inactivation of inflammatory responses of macrophages by pMSC-CM. The expression of a panel of monocyte-/macrophage-expressing gene transcripts (total seventy-seven, as seen in the left column of the table) in THP1 cells from three different groups (control, PMA/LPS, and PMA/LPS+50% pMSC-CM) was examined by using the PCR array following the manufacturer's instruction (SABiosciences—QIAGEN Inc., Valencia, CA, USA), and each group represented three separate samples. In brief, 1 *μ*g of high-quality total RNA from each sample was reverse transcribed to cDNA by using the RT^2^ First Strand Synthesis Kit (QIAGEN). The expression of the selected genes was then amplified by real-time PCR using RT^2^ Profile PCR arrays with PCR amplification conditions (10 min at 95°C, followed by 45 cycles of 15 s at 95°C and 60 s at 60°C) according to the manufacturer's instruction (QIAGEN Inc.). The fold change of each target transcript and statistical comparison between groups were determined by using the manufacturer's online web analysis tools (http://www.sabiosciences.com/pcrarraydataanalysis.php). A negative number indicated a fold decrease, whereas a positive number implied a fold increase.
