# Supplementary material for: Protection of the Peritoneal Membrane by Peritoneal Dialysis Effluent-Derived Mesenchymal Stromal Cells in a Rat Model of Chronic Peritoneal Dialysis
Source: Stem Cells Int. 2019 Sep 16;2019:8793640. doi: 10.1155/2019/8793640 (PMC6766137; doi:10.1155/2019/8793640)
Supplement: Supplementary Materials — Supplementary Table 1: inactivation of inflammatory responses of macrophages by pMSC-CM. The expression of a panel of monocyte-/macrophage-expressing gene transcripts (total seventy-seven, as seen in the left column of the table) in THP1 cells from three different groups (control, PMA/LPS, and PMA/LPS+50% pMSC-CM) was examined by using the PCR array following the manufacturer's instruction (SABiosciences—QIAGEN Inc., Valencia, CA, USA), and each group represented three separate samples. In brief, 1 μg of high-quality total RNA from each sample was reverse transcribed to cDNA by using the RT2 First Strand Synthesis Kit (QIAGEN). The expression of the selected genes was then amplified by real-time PCR using RT2 Profile PCR arrays with PCR amplification conditions (10 min at 95°C, followed by 45 cycles of 15 s at 95°C and 60 s at 60°C) according to the manufacturer's instruction (QIAGEN Inc.). The fold change of each target transcript and statistical comparison between groups were determined by using the manufacturer's online web analysis tools (http://www.sabiosciences.com/pcrarraydataanalysis.php). A negative number indicated a fold decrease, whereas a positive number implied a fold increase. [file 8793640.f1.pdf]

Supplementary Table 1: Inactivation of inflammatory responses of macrophages by pMSCs-CM

Affected gene transcripts	Fold changes by PMA/LPS (vs. Control)	P value (PMA/LPS vs. Control, n = 3)	Fold changed by pMSC-CM (vs PMA/LPS)	P value (PMA/LPS vs. PMA/LPS + 50%, n = 3)
CXCL8	535.95	**0.009573**	-9.14	0.083365
IL1B	500.8	**0.048456**	-6.25	0.123176
CXCL6	242.74	**0.001276**	-10.08	**0.032372**
CXCL1	235.55	0.116816	-5.27	0.247662
CXCL3	190.02	**0.038563**	-2.32	0.133338
IL1RN	177.15	**0.004863**	-4.02	**0.037209**
CXCL2	92.93	0.128867	-12.97	0.153547
CXCL5	78	0.09673	-6.37	0.401279
IL6	46.15	0.199307	-2.13	0.476512
CCL4	33.91	0.118846	-1.81	0.447106
IL1A	20.08	0.109787	-3.51	0.26583
NOS2	11.17	**0.007915**	-9.35	**0.008181**
PTGS2	9.8	0.106712	-2.33	0.302766
LY96	7.56	0.152586	-1.42	0.521316
CCL2	6.83	0.122509	-4.45	0.110069
RIPK2	6.02	**0.013444**	-5.35	0.058044
BCL6	5.27	**0.029664**	-2.39	0.085688
CSF1	4.9	0.080272	-1.88	0.270602
CCL3	4.12	0.224417	-2.59	0.273153
CCL7	4.01	0.209097	-2.64	0.251111
CCL5	3.8	0.065136	-8.77	**0.01768**5
ITGB2	2.97	**0.036245**	-2.54	0.056093
NR3C1	2.45	0.234157	-7.81	**0.043945**
IL1R1	2.25	0.147725	-1.47	0.349567
MYD88	2.82	0.092337	-4.90	0.058324
TNF	1.98	0.052401	-1.46	0.140847
CEBPB	1.75	0.226914	-1.71	0.089656
CD14	1.68	0.268575	-1.03	0.593546
C3	1.35	0.284091	1.39	0.106648
C3AR1	-1.53	0.268667	1.07	0.593999
CCL11	-1.5	0.304463	1.73	0.069919
CCL13	1.25	0.489838	-1.26	0.173406
CCL16	-1.2	0.665358	1.69	0.108011
CCL17	-1.12	0.936866	1.68	0.186882
CCL19	-1.16	0.700046	1.82	0.142731
CCL21	-1.21	0.565338	1.24	0.466697
CCL22	1.27	0.185247	1.22	0.24265
CCL23	-1.79	0.327176	1.02	0.871449
CCL24	-1.12	0.575359	1.33	0.129974
CCL8	-1.26	0.548401	1.51	0.176782

CCR1	-1.27	0.428263	1.74	**0.026517**
CCR3	-1.43	0.4268	1.92	0.065713
CCR4	1.18	0.48383	1.92	**0.01117**
CCR7	1.05	0.887337	1.51	0.100115
CD40LG	-1.48	0.373571	1.39	0.397193
CRP	-1.07	0.929725	1.51	0.273909
CXCL10	-1.09	0.610765	-2.76	0.312984
CXCL9	-1.67	0.138019	1.97	**0.000092**
CXCR1	-1.11	0.662785	1.60	**0.035095**
CXCR2	-1.29	0.430072	1.49	0.109703
FASLG	-1.38	0.373761	1.51	0.091681
FOS	1.18	0.524644	1.37	0.256317
IL10	-1.38	0.309707	1.93	**0.015066**
IL10RB	1.24	0.285129	-1.19	0.366473
IL18	-1.16	0.622399	-1.29	0.303259
IL1RAP	1.05	0.916089	1.03	0.851851
IL23A	1.12	0.851924	1.60	**0.016877**
IL6R	-1.36	0.179679	1.65	**0.00420**5
IL9	-1.25	0.522482	1.93	**0.024161**
KNG1	-1.25	0.320858	1.80	**0.01354**
LTA	-1.02	0.933977	1.53	0.197138
LTB	1.11	0.711759	1.24	0.311127
NFKB1	-1.23	0.454782	1.18	0.299521
SELE	-1.2	0.477778	1.86	**0.005707**
TIRAP	-1.04	0.820124	1.24	0.283465
TLR1	1.01	0.991327	1.34	0.24833
TLR2	1.23	0.73762	-1.72	0.183617
TLR3	-1.26	0.363668	2.17	**0.008944**
TLR4	-1.09	0.65828	-1.13	0.421518
TLR5	-1.38	0.298214	1.86	**0.036134**
TLR6	-1.24	0.410177	1.25	0.098511
TLR9	1.13	0.653286	1.50	0.20164
TNFSF14	1.09	0.63339	1.49	0.177894
TOLLIP	1.3	0.325699	1.13	0.740054
CXCR4	-2.14	0.127673	1.36	0.350269
CCR2	-2.42	0.076988	1.82	0.11419
TLR7	-8.43	0.285805	-1.02	0.833967

The regulatory effects of pMSCs-CM on expression of inflammation-related seventy-seven gene transcripts in PMA/LPS-activated THP1 cells was examined by using PCR array. The fold change of each transcript level in experimental group as compared to control group (PMA/LPS vs. Control, or PMA/LPS + 50% pMSCs-CM vs. PMA/LPS) was presented in mean of three separate RNA samples (n = 3). – (negative number) represented downregulation. A P value of less than 0.05 was highlighted in bold

Supplementary Table 1: Inactivation of inflammatory responses of macrophages by pMSCs-CM

The expression of a panel of monocyte/macrophage-expressing gene transcripts (total seventy-seven, as seen in the left column of the table) in THP1 cells from three different groups (Control, PMA/LPS, and PMA/LPS + 50% pMSCs-CM) was examined by using PCR array following manufacturer's instruction (SABiosciences – QIAGEN Inc., Valencia, CA, USA), and each group represented three separate samples. In brief, 1 μg of high quality total RNA from each sample was reverse transcribed to cDNA by using RT^2 First Strand Synthesis Kit (QIAGEN). The expression of selected genes was then amplified by real-time PCR using RT^2 Profile PCR arrays with PCR amplification conditions (10 min at 95°C, followed by 45 cycles of 15 s at 95°C and 60 s at 60°C) according to manufacturer's instruction (QIAGEN Inc.). The fold change of each target transcript and statistical comparison between groups were determined by using the manufacturer's online web analysis tools (www.SABiosciences.com/pcrarraydataanalysis.php.). A negative number indicated a fold decrease, whereas a positive number implied a fold increase.
